# Supplementary material for: The Safety and Efficacy of Psychosocial Adherence Interventions in Young People with Early Psychosis: A Systematic Review
Source: Healthcare (Basel). 2022 Sep 9;10(9):1732. doi: 10.3390/healthcare10091732 (PMC9498603; doi:10.3390/healthcare10091732)
Supplement: Supplementary file 1 [file healthcare-10-01732-s001.zip › healthcare-1865682-supplementary.pdf]

Supplementary File S1: Search Terms

The following search strategy will employ (keywords are italicized; subject headings are emboldened; Boolean operators are capitalized):

1. compliance/compliances"[All Fields] OR "patient compliance"[MeSH Terms] OR ("patient"[All Fields] AND "compliance"[All Fields]) OR "patient compliance"[All Fields] OR "compliance"[All Fields] OR "compliance"[MeSH Terms]
2. adherence/adherence"[All Fields] OR "adhere"[All Fields] OR "adhered"[All Fields] OR "adherence"[All Fields] OR "adherences"[All Fields] OR "adherent"[All Fields] OR "adherents"[All Fields] OR "adherer"[All Fields] OR "adherers"[All Fields] OR "adheres"[All Fields] OR "adhering"[All Fields]
3. concordance/ concordance"[All Fields] OR "concordances"[All Fields] OR "concordancy"[All Fields] OR "concordant"[All Fields]
4. early psychosis/ "psychotic disorders"[MeSH Terms] OR ("psychotic"[All Fields] AND "disorders"[All Fields]) OR "psychotic disorders"[All Fields] OR "psychosis"[All Fields]
5. psychosocial interventions/ psychosocial intervention"[MeSH Terms] OR ("psychosocial"[All Fields] AND "intervention"[All Fields]) OR "psychosocial intervention"[All Fields] OR ("psychosocial"[All Fields] AND "interventions"[All Fields]) OR "psychosocial interventions"[All Fields]
6. "complia* OR adhere* OR concord*" ("compliances"[All Fields] OR "patient compliance"[MeSH Terms] OR ("patient"[All Fields] AND "compliance"[All Fields]) OR "patient compliance"[All Fields] OR "compliance"[All Fields] OR "compliance"[MeSH Terms] OR ("adherance"[All Fields] OR "adhere"[All Fields] OR "adhered"[All Fields] OR "adherence"[All Fields] OR "adherences"[All Fields] OR "adherent"[All Fields] OR "adherents"[All Fields] OR "adherer"[All Fields] OR "adherers"[All Fields] OR "adheres"[All Fields] OR "adhering"[All Fields]) OR ("concordance"[All Fields] OR "concordances"[All Fields] OR "concordancy"[All Fields] OR "concordant"[All Fields]))
7. ("early"[All Fields] AND ("psychotic disorders"[MeSH Terms] OR ("psychotic"[All Fields] AND "disorders"[All Fields]) OR "psychotic disorders"[All Fields] OR "psychosis"[All Fields]))
8. ("psychosocial intervention"[MeSH Terms] OR ("psychosocial"[All Fields] AND "intervention"[All Fields]) OR "psychosocial intervention"[All Fields] OR ("psychosocial"[All Fields] AND "interventions"[All Fields]) OR "psychosocial interventions"[All Fields]))
9. 6 AND 7 AND 8
